# Evaluation of Sourdough Bread and Its Potential Use in Support of the Treatment of Chronic Non-Communicable Diseases

**DOI:** 10.3390/nu16152485

**Published:** 2024-07-31

**Authors:** Adrian Bartos, Alicja Malik, Anna Diowksz, Grażyna Podolska, Joanna Leszczyńska

**Affiliations:** 1 Department of Bioinorganic Chemistry, Faculty of Pharmacy, Medical University of Lodz, Jana Muszyńskiego 1, 90-151 Łódź, Poland; 2 Institute of Natural Products and Cosmetics, Faculty of Biotechnology and Food Sciences, Łódź University of Technology, Stefanowskiego 2/22, 90-537 Łódź, Poland; alicja.malik@p.lodz.pl; 3 Institute of Fermentation Technology and Microbiology, Faculty of Biotechnology and Food Sciences, Łódź University of Technology, Wólczańska 171/173, 90-530 Łódź, Poland; anna.diowksz@p.lodz.pl; 4 Institute of Soil Science and Plant Cultivation, Czartoryskich 8, 24-100 Puławy, Poland; aga@iung.pulawy.pl

**Keywords:** bread, gluten, fermentation, immunoreactivity, angiotensin, alpha amylase

## Abstract

Gastrointestinal disorders dysregulate the biochemical environment of the gastrointestinal tract by altering pH conditions during the gastric phase of digestion or by reducing the secretion of pancreatin during the intestinal part of the process. Ingested functional food could therefore lose some of its health-promoting potential apart from its nutritional value. In this work, we aimed to manufacture bread marked by decreased gluten content, using a commercial or laboratory sourdough, that could be appropriate for patients afflicted with wheat allergy, hypertension and pancreatic malfunctions. A reference sample (no sourdough) was prepared alongside wheat and wheat–rye bread samples—produced with either commercial or laboratory sourdough (*L. plantarum* BS, *L. brevis* 1269, *L. sanfranciscensis* 20663). We measured the QQQPP allergen content (ELISA) in bread extracts digested in vitro and determined how these extracted components affect the level of active angiotensin and alpha amylase (spectrophotometry). We then elucidated how these properties changed when physiological digestion conditions (pH and pancreatin activity) were disturbed to mimic gastric hyperacidity, hypochlorhydria or exocrine pancreatic insufficiency. The key finding was that every tested type of bread produced with laboratory sourdough exhibited pronounced angiotensin-converting enzyme inhibition. The effect was preserved even in dysregulated digestive conditions. The use of laboratory sourdough prevented an increase in allergenicity when pancreatin was restricted as opposed to the commercial sourdough, which surpassed the reference sample reading at 50% pancreatin. No statistically consistent link was reported when the inhibition of alpha amylase was assayed. In conclusion, functional bread manufactured with sourdough composed of *L. plantarum* BS, *L. brevis* 1269, and *L. sanfranciscensis* 20663 was shown to be potentially capable of contributing to the treatment against hypertension as evidenced by in vitro research. It was also moderately safer with regard to its allergenicity.

## 1. Introduction

Gluten is a family of structural and storage proteins found in cereals such as wheat, barley and rye [1,2,3]. It makes up 85% of the total grain protein [4]. Gluten is composed of two main fractions—prolamins and glutelins [5]. Specifically in the case of wheat, these two fractions are referred to as gliadins and glutenins, respectively [6]. In barley, it is hordein and hordenin, respectively [7,8]. Prolamins in rye are known as secalins [9]. Gliadins are the underlying factor behind wheat allergy [10,11], while prolamins as a whole evoke the gastrointestinal disorder known as coeliac disease (also known as gluten-sensitive enteropathy) [12]. To minimize the exposure of these at risk, efforts are made to somewhat degrade gliadin at an early manufacturing stage of dough manufacturing [13] or fermentation [14] or to facilitate the digestion of gluten with an enzyme introduced to the gastrointestinal tract [15]. Otherwise, adherence to a gluten-free diet is the main resort in defense against associated symptoms [16]. Such a solution is however imperfect due to gluten contamination arising from cross-contact [17] and nutritional deficiencies in patients caused by the elimination diet [18,19]. Scientific literature hints that gluten content can be minimized with the use of gliadin-degrading microbiota used at the stage of sourdough fermentation [20].

In this report, we hypothesize that the impact of sourdough fermentation reaches beyond the possibility of reducing the content of gluten and that it could also affect three other properties of bread: its immunogenicity, the ability to inhibit the conversion of angiotensin and the ability to inhibit α-amylase. Using a simulated digestion procedure, we present the outcome determined in the physiological state of the digestive system. Ultimately, a minor adjustment made to the process of dough fermentation could suit the needs of coeliac patients alongside these afflicted by wheat allergy, hypertension or diabetes.

Whether its gluten content is high or low, ingested bread is invariably subjected to low pH conditions in the stomach as well as enzymatic decomposition in the intestine [21]. In some cases, parameters associated with these processes deviate from physiological values and tend to be dysregulated depending on the gastrointestinal health of the consumer. Such deviations, as those described below, could additionally affect the properties of bread and the effect it exerts on the human body. This includes cases when either pH (hyperacidity, hypochlorhydria) or pancreatin secretion (exocrine pancreatic insufficiency) is disturbed.

Gastric hyperacidity (acid dyspepsia) occurs when the concentration of hydrochloric acid in the stomach is abnormally high. This can lead to irritation of the mucous membrane lining the inner layer of the stomach or contribute to gastroesophageal reflux disease [22]. In the latter case, a reflux of excess acid into the esophagus is responsible for numerous adverse symptoms, like heartburn, dysphagia and a group of atypical symptoms afflicting the throat, larynx or lungs [23,24]. Hyperacidity may also be involved in the development of chronic renal failure [25].

Contrary to gastric hyperacidity, hypochlorhydria (or gastric hypoacidity) is a disorder marked by the deficiency of stomach acid [26]. A low concentration of hydrochloric acid entails elevated pH, which in turn leads to indigestion, malnutrition, greater vulnerability to pathogens introduced with food and a list of atypical symptoms afflicting the gastrointestinal tract [27,28]. Hypochlorhydria may be conducive to the development of duodenal dysbiosis [29] and hypergastrinemia [30].

Another case of the dysregulated digestive system is associated with pancreatin, the activity of which is routinely decreased in the course of a condition known as exocrine pancreatic insufficiency [31]. It underlies the manifestation of symptoms similar to functional gastrointestinal disorders (including irritable bowel syndrome, functional dyspepsia, or functional constipation) [32,33].

Having performed an investigation of the physiological conditions of the gastrointestinal system, we therefore took into consideration the effect exerted by bread under these three deviated conditions with variable chemical and biochemical backgrounds. This work is a continuation and a pivotal extension of preliminary research published a few years ago [34]. It is advantageous in that it identifies the exact microbial composition of sourdough (three bacterial strains selected from the pool of 35 strains tested) and links the products thereof to the scientific findings on clinical food safety. The scientific gap it undertakes to address is to elucidate the health-promoting properties of bread in conjunction with two other vital aspects that are routinely omitted in these kinds of studies. Namely, we emphasized the role of bacterial monitoring and identification that precedes the actual food evaluation. Other than that, our study acknowledges the variable conditions of the gastrointestinal environment. An investigation confined to standard values is not always accurate, as it does not reflect an actual differentiation reported in clinical practice.

## 2. Materials and Methods

### 2.1. Pre-Selection of Bacterial Strains

In order to select the most suitable bacterial strains for the bread fermentation process, the gliadin-digesting capabilities of the 35 bacterial strains were tested. These included *Lactobacillus amylovorus* 20532, *Lactobacillus sanfranciscensis* 20663, *Lactobacillus plantarum* 98/L.p., *Lactobacillus brevis* 99/L.b., *Lactobacillus sanfranciscensis* 94/L.S., *Lactobacillus brevis* 1269, *Lactobacillus brevis* 1267, *Lactobacillus paracasei* LOCK 0924, *Lactobacillus paracasei* LOCK 0910, *Lactobacillus rhamnosus* LOCK 1089, *Lactobacillus casei* LOCK 0919, *Lactobacillus casei* LOCK 1091, *Lactobacillus paracasei* LOCK 0922, *Lactobacillus casei* LOCK 0915, *Lactobacillus casei* LOCK 0911, *Lactobacillus rhamnosus* LOCK 1087, *Lactobacillus rhamnosus* LOCK 1088, *Lactobacillus curvatus* 9M, *Lactobacillus curvatus* 7M, *Lactobacillus curvatus* 4M, *Lactobacillus curvatus* 1M, *Lactobacillus fermentum* T98, *Lactobacillus fermentum* T53, *Lactobacillus brevis* 2351, *Lactobacillus brevis* 99, *Lactobacillus brevis* 18M, *Lactobacillus brevis* PCM 488, *Lactobacillus sanfranciscensis* L0866, *Lactobacillus plantarum* syn. *cucumoris* L0865, *Lactobacillus plantarum* BS, *Lactobacillus plantarum* B01149, *Lactobacillus plantarum* L0862, *Lactobacillus plantarum* L0861, *Lactobacillus plantarum* L0860, *Lactobacillus plantarum* L0859, and *Lactobacillus brevis* 3M.

A 24 h inoculum of each bacterial strain was prepared as a starter culture at 10^9^ cfu/cm^3^. Bread dough samples were composed of 100 g of type 500 flour (containing 18.8% gluten, 0.51% ash and 14.9% water), 100 g water and 2% bacterial inoculum. The samples were subject to fermentation at 30 °C for 24 h, which was followed by termination of the process performed by incubation at 100 °C for 10 min.

**Figure 1:**
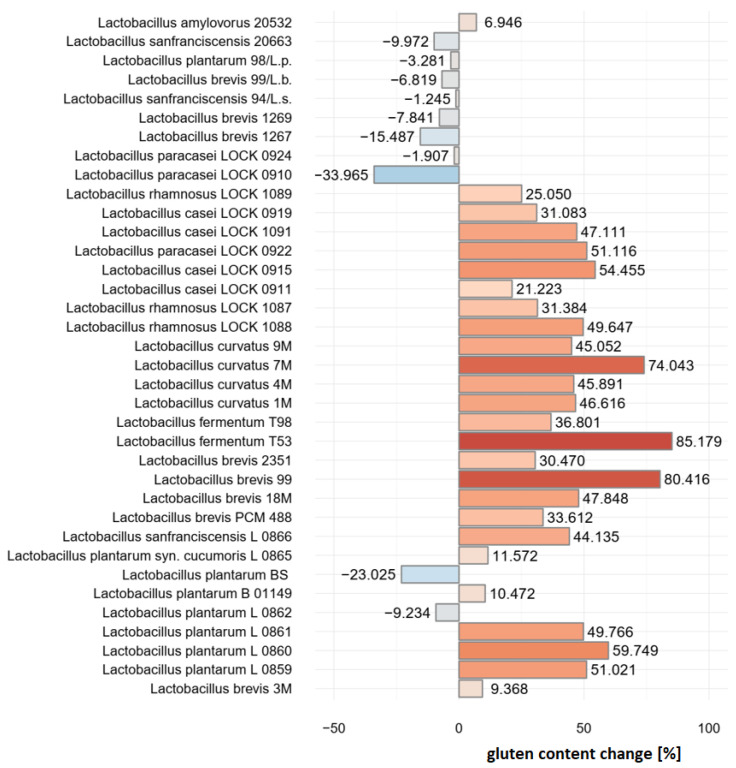
Bacterial strains used as inoculum that caused decrease in gliadin [%] relative to the non-fermented reference bread sample.

As many as 26 strains were shown to increase the antigenicity of gliadin [%] relative to the reference bread sample that was not subjected to lactic acid fermentation (not inoculated). The mean increase reported among these strains was 41.53%. The other 10 strains were identified as those that cause a decrease in the antigenicity of gliadin with an overall mean of 12.28%. The results are presented in Figure 1 below. The degradation of gliadin was confirmed with MALDI-TOF MS.

Strains *L. plantarum* BS, *L. brevis* 1269 and *L. sanfranciscensis* 20663 were selected as the most optimal inoculum components based on their superior stability compared to the other 6 strains.

### 2.2. Sample Preparation

Upon the selection of the three most efficient bacterial strains at gliadin degradation, the following types of bread samples were prepared in-house for research purposes:

(A.) Reference bread (no sourdough)

Ingredients and preparation: 300 mL warm water (ca. 40 °C), 10 g table salt, 500 g wheat flour, 15 g table sugar, and 5 g baker’s yeast. The dough was kneaded and mixed with 15 g of cooking oil. Three rising steps were carried out—1 h followed by dough mixing, 30 min and lastly, 40 min rising of dough divided into 55 g pieces. Dough was baked at 220 °C with initial steaming for 5 min.

(B.) Wheat sourdough bread I

Ingredients and preparation: 350 mL warm water (ca. 40 °C), 10 g table salt, 500 g wheat flour, commercial wheat sourdough with yeast (Browin, Łódź, Poland, product no. 409431). The dough was kneaded and mixed with 15 g of cooking oil. It was left to rise for 1 h rise at room temperature before it was baked at 220 °C with initial steaming for 5 min.

Wheat sourdough packet contents: dried wheat leaven 65.2% (ground wheat products, wheat malt, leaven starter cultures), active dry yeast 34.8% (*S. cerevisiae*), vegetable oil, gum arabic (E414), ascorbic acid (E300), sorbitan monostearate (E491).

(C.) Wheat–rye sourdough bread I

Ingredients and preparation: 350 mL warm water (ca. 40 °C), 10 g table salt, 350 g wheat flour, 150 g rye flour, commercial rye sourdough with yeast and malt (Browin, Łódź, Poland, product no 409421). The dough was kneaded and mixed with 15 g of cooking oil. It was left for 1 h to rise at room temperature before it was baked at 220 °C with initial steaming for 5 min.

Wheat sourdough packet contents: dried rye sourdough 65.2% (ground rye products, barley malt extract), active dry yeast 34.8% (*S. cerevisiae*), vegetable oil, gum arabic (E414), ascorbic acid (E300), sorbitan monostearate (E491).

(D.) Wheat sourdough bread II

Ingredients and preparation: 98 g warm water (ca. 40 °C), 10 g table salt, 260 g wheat flour, 240 g sourdough (*L. plantarum* BS, *L. brevis* 1269, *L. sanfranciscensis* 20663) prepared in-house at the Institute of Fermentation Technology and Microbiology, 5 g baker’s yeast, 15 g table sugar. The dough was kneaded and mixed with 15 g of cooking oil. Three rising steps were carried out—1 h followed by dough mixing, 30 min and lastly, 40 min rising of dough divided into 55 g pieces. Dough was baked at 220 °C with initial steaming for 5 min.

Sourdough was prepared with 150 g wheat flour, 150 g water and 2 mL of 24 h inoculum of each bacterial strain and then subjected to fermentation at 30 °C for 24 h.

(E.) Wheat–rye sourdough bread II

Ingredients and preparation: 98 g warm water (ca. 40 °C), 10 g table salt, 130 g wheat flour, 130 g rye flour, 240 g sourdough (*L. plantarum* BS, *L. brevis* 1269, *L. sanfranciscensis* 20663) prepared in-house at the Institute of Fermentation Technology and Microbiology, 5 g baker’s yeast, 15 g table sugar. The dough was kneaded and mixed with 15 g of cooking oil. Three rising steps were carried out—1 h followed by dough mixing, 30 min and lastly, 40 min rising of dough divided into 55 g pieces. Dough was baked at 220 °C with initial steaming for 5 min.

Sourdough was prepared with 150 g wheat flour, 150 g water and 2 mL of 24 h inoculum of each bacterial strain and then subjected to fermentation at 30 °C for 24 h.

### 2.3. Simulated Digestion

Prior to further analysis, bread samples were subjected to the simulated digestion procedure performed to differentiate two variables: pH conditions during the gastric phase of the process and the extent of exposure to pancreatin (100%, 75% and 50% enzyme activity, where a dose of 200 mg accounted for 100% pancreatin activity) during the intestinal phase of digestion.

Conditions used for simulating gastrointestinal disorders:

- pH = 1 (strong gastric hyperacidity);
- pH = 2 (gastric hyperacidity);
- pH = 3 (physiological conditions);
- pH = 4 (gastric hypoacidity / hypochlorhydria);
- pancreatin at 100% activity (physiological conditions);
- pancreatin at 75% activity (exocrine pancreatic insufficiency);
- pancreatin at 50% activity (strong exocrine pancreatic insufficiency).

List of chemicals and enzymes used:

- 0.3 M calcium chloride;
- α-amylase from porcine pancreas (EC 3.2.1.1), type Vi-B, ≥10 units/mg solid;
- Pepsin from porcine gastric mucosa, powder, ≥250 units/mg solid (Sigma-Aldrich, St. Louis, MO, USA);
- Bile bovine, dried Ox gall powder (Sigma-Aldrich);
- Pancreatin (Kreon travix, 10000, Abbott Products GMBH, Wiesbaden, Germany);
- 1 M hydrochloric acid;
- 1 M sodium hydroxide.

Procedure description:

(a.) Salivary phase: First, 0.7 mL SSF, prepared as described in Table 1, was mixed with 295 µL H_2_O and 5 µL 0.3 M CaCl_2_. The solution was then used to dissolve 4.69 mg α-amylase, which was followed by the addition of 1 g sample, which was prepared as described in Section 2.2. The mixture was incubated for 2 min at 37 °C.

(b.) Gastric phase: Afterwards, 1.5 mL SGF, prepared as described in Table 2, was mixed in a fresh test tube with 0.459 µL H_2_O, 1 µL 0.3 M CaCl_2_ and 40 µL 1 M HCl. The solution was then used to dissolve 12 mg pepsin, which was followed by addition of the analytical test sample pre-treated as described above. Four individual samples were prepared where the pH value was set to 1, 2 (both to simulate gastric hyperacidity), 3 (physiological pH upon food consumption) and 4 (to simulate gastric hypoacidity), respectively, using 6 M HCl. The samples were incubated for 2 h at 37 °C.

(c.) Intestinal phase: In a fresh test tube, 2.2 mL SIF, prepared as described in Table 3, was mixed with 0.5 mL bile, 1.262 mL H_2_O, 8 µL 0.3 M CaCl_2_, 30 µL 1 M NaOH and 200 mg pancreatin. Analytical test samples, each at a distinct pH value, prepared and pre-treated as described above, were added to the solution, and the pH was set to 7 using 1 M NaOH in every sample. The samples were incubated for 2 h at 37 °C. In addition, an analytical test sample previously digested at physiological pH = 3 was not only subjected to 200 mg pancreatin (100% activity) but also to lower doses of the enzyme, which was thought to mimic the treatment with 75% and 50% pancreatin activity.

### 2.4. QQQPP Peptide Determination with ELISA

The analysis was carried out with the use of the following reagents:

- PBS buffer (0.7551 g KH_2_PO_4_; 11.466 g Na_2_HPO_4_ and 18 g NaCl dissolved in 2 L distilled water);
- Carbonate-bicarbonate buffer;
- PBS-T buffer (1 mL Tween per every 1 L PBS);
- Milk powder 3% solution in PBS (3 g milk powder in 1 mL PBS);
- Rabbit serum with anti-QQQPP antibodies;
- Anti-Rabbit antibodies (Anti-Rabbit IgG (whole molecule)–peroxidase, Sigma-Aldrich);
- TMB (3,3′,5,5′-tetramethylbenzidine) Liquid Substrate System for ELISA, Sigma-Aldrich);
- 1 M sulfuric acid.

Bread sample extracts were prepared over 1 h incubation at room temperature of a respective 0.5 g bread sample, as described in Section 2.2, and 5 mL water. Upon 10 min centrifugation at 5000 rpm, sample extracts were diluted 1000-fold and used to coat a 96-well microplate. Wells designated as negative controls were coated with 3% milk powder solution. The plate was incubated overnight at 4 °C. The following day, the coating solutions were discarded, and the plate was washed 4 times with PBS-T. Unbound sites at the wells were blocked with 3% milk powder solution. The plate was incubated for 2 h at room temperature. Afterwards, it was washed 4 times with PBS-T.

Rabbit sera containing anti-QQQPP peptide antibodies were diluted 100-fold and applied to the plate at 100 µL per well. The plate was incubated for 1 h at room temperature and then washed 4 times with PBS-T. Anti-rabbit IgG–peroxidase antibody produced in goat were diluted 1000-fold and applied to the plate at 100 µL per well. Again, the plate was incubated for 1 h at room temperature and then washed 4 times with PBS-T. TMB substrate was applied to the plate at 100 µL per well and incubated 30–60 min at room temperature. The reaction was terminated in each well using 100 µL 1 M H_2_SO_4_. The absorbance was measured at a wavelength of 450 nm with a microplate reader. The results were calculated based on a standard curve prepared with a serially diluted QQQPP peptide solution.

### 2.5. Inhibition of Enzymatic Conversion of Angiotensin

The analysis was carried out with the use of the following reagents:

- Sodium-potassium buffer at pH 8.3 (0.2 M K_3_PO_4_ and 0.3 M NaCl);
- N-Hippuryl-His-Leu hydrate, HHL (Sigma-Aldrich);
- Angiotensin-converting enzyme from rabbit lung, ACE (Sigma-Aldrich);
- 1 M hydrochloric acid;
- Ethyl acetate.

Bread sample extracts were prepared over 1 h incubation at room temperature of a respective 0.5 g bread sample, as described in Section 2.2, and 5 mL water. Upon 10 min centrifugation at 5000 rpm, the volume of the 15 µL extract sample was then added to 110 µL 10 mM HHL (dissolved in sodium–potassium buffer) and 26 mU ACE (dissolved in 50% glycerol) per mL of reaction medium. Upon 80 min incubation at 37 °C, 110 µL 1 M hydrochloric acid was used to disable enzymatic activity. The reaction product was extracted with 1 mL ethyl acetate. The volume of the 750 µL organic phase was collected and dried for 15 min at 90 °C. Dry substance was resuspended in 1 mL distilled water. Absorbance was taken at 228 nm.

In addition to analytical samples described above, a control sample and two blank samples were prepared:

- Negative control: 15 µL extract sample was replaced with 15 µL water to prevent enzymatic reaction due to the lack of substrate;
- Reaction blank: HCl was added before ACE so that a 15 µL extract sample could not be converted enzymatically;
- Sample blank: 15 µL extract sample was replaced with 15 µL water; HCl was added before ACE.

Calculations:

Absorbance values, measured as described above, were used to evaluate the inhibition of enzymatic conversion of angiotensin in line with the following formula:

$$\%\;IACE=100a\cdot \frac{\left(A-B\right)-\left(C-D\right)}{A-B}$$

*A*—negative control;
*B*—reaction blank;
*C*—analytical sample;
*D*—sample blank.

### 2.6. Inhibition of α-Amylase

The analysis was carried out with the use of the following reagents:

- Phosphate buffer pH 7.0, 0.1 M (61.5 mL 1 M K_2_HPO_4_ solution mixed with 38.5 mL 1 M KH_2_PO_4_ solution and filled up with water to final volume 200 mL);
- α-amylase solution: 100 mg α-amylase from porcine pancreas, EC 3.2.1.1 (type Vi-B, ≥10 units/mg solid) dissolved in 400 mL distilled water;
- Starch solution: 0.125 g starch dissolved in 25 mL phosphate buffer pH 7.0 and incubated for 20 min at 65 °C;
- DNS solution: 1 g 3,5-dinitrosalicylic acid and 30 g potassium sodium tartrate were dissolved in 20 mL 2 M NaOH. The solution was filled up to 100 mL with distilled water.

Bread sample extracts were prepared over 1 h incubation at room temperature of a respective 0.5 g bread sample, as described in Section 2.2, and 5 mL water. Upon 10 min centrifugation at 5000 rpm, 100 µL extract was mixed with 500 µL α-amylase solution and 500 µL phosphate buffer. The mixture was incubated for 10 min at room temperature. Afterwards, 500 µL starch solution was added, which was followed by 10 min incubation at room temperature. Lastly, 1 mL DNS solution was added, which was followed by 5 min incubation in a water bath at 95 °C. Samples were cooled and filled up with distilled water to the final volume of 10 mL. Absorbance was taken at 540 nm.

### 2.7. Statistical Analysis

Statistical significance was calculated using Shapiro–Wilk, Brown–Forsythe and Kruskal–Wallis tests performed on Statistica software version 13.3 and a two-way ANOVA test performed on GraphPad Prism software version 8.3.0. *p*-values of <0.05 were considered significant.

## 3. Results

This section was divided into three categories wherein distinct bread properties are determined: immunoreactivity (allergenic QQQPP peptide determination), inhibition of enzymatic conversion of angiotensin (IACE), inhibition of α-amylase. Each of these consists of two sub-categories where results were collected under different digestive conditions, i.e., pH in the range of 1–4 (gastric disorders), or the activity of pancreatin at 100% (enzyme dose as in standard digestion conditions, 200 mg) as well as reduced activity of pancreatin (75% and 50% of the standard dose), which is referred to as intestinal disorders. Both these variables are hypothesized to affect the tested properties of bread and the outcome of its ingestion. At the same time, they are thought to represent alterations in the gastrointestinal biochemical environment caused by disorders such as, for instance, gastric hyperacidity and hypochlorhydria, or exocrine pancreatic insufficiency. Afflicted patients may therefore exhibit a varied extent of tolerance and manifestation of symptoms, or they may incompletely benefit from the health-promoting effects from the consumed food.

### 3.1. Immunoreactivity

**Figure 2:**
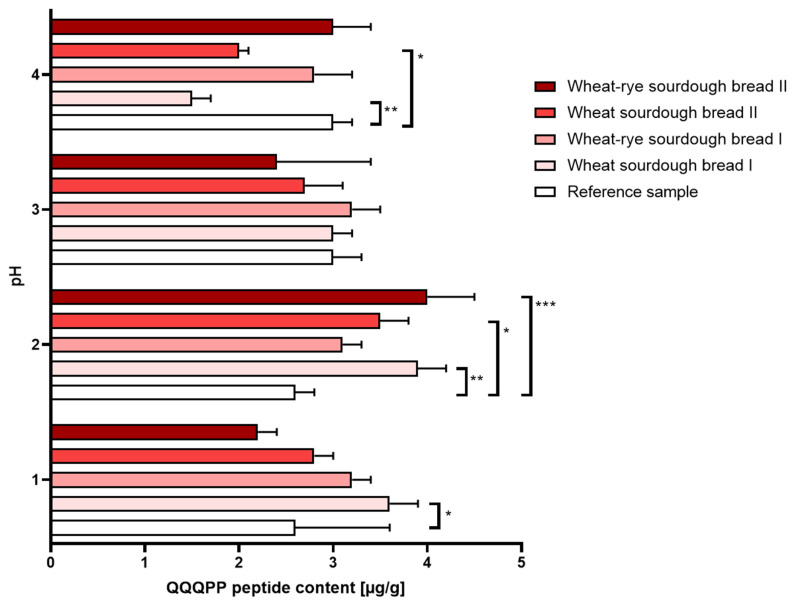
Immunoreactivity of gluten (±SD) determined in bread samples digested under different pH conditions corresponding to: hyperacidity (pH = 1 and 2), physiological pH = 3 upon food consumption and hypoacidity at pH = 4. Statistical significance by 2-way ANOVA at * *p* = 0.0215, 95% CI of diff. −1.879 to −0.1207 (pH = 1); * *p* = 0.0435, 95% CI of diff. −1.779 to −0.02065 (pH = 2); * *p* = 0.0218, 95% CI of diff. 0.1184 to 1.882; ** *p* = 0.021, 95% CI of diff. −2.179 to −0.4207 (pH = 2); ** *p* = 0.0015, 95% CI of diff. 0.5144 to 2.486 (pH = 4); *** *p* = 0.0009, 95% CI of diff. −2.279 to −0.5207.

**Figure 3:**
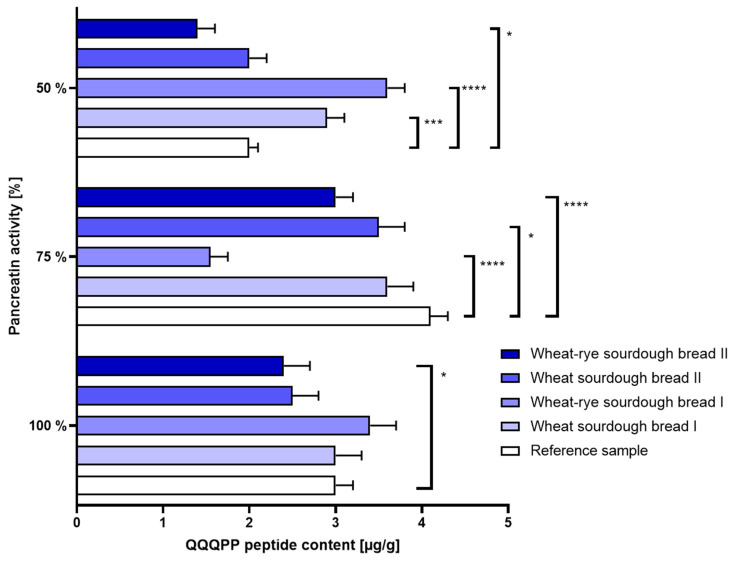
Immunoreactivity of gluten (±SD) determined in bread samples digested under different enzymatic conditions corresponding to 100%, 75% and 50% activity of pancreatin during intestinal phase of simulated digestion. Statistical significance by 2-way ANOVA at * *p* = 0.0165, 95% CI of diff. 0.09301 to 1.107; *** *p* = 0.0003, 95% CI of diff. −1.407 to −0.3930; **** *p* < 0.0001, 95% CI of diff. at 75% pancreatin: 2.043 to 3.057 (reference vs. wheat–rye sourdough bread I), 0.5930 to 1.607 (reference vs. wheat–rye sourdough bread II), 95% CI of diff. at 50% pancreatin: −2.107 to −1.093.

(a.) Gastric disorders: Digestion performed under the conditions mimicking gastric hypoacidity resulted in a decreased immunoreactivity reported in wheat bread regardless of the sourdough used for manufacturing. Gastric hyperacidity, on the other hand, was mostly characterized by an increase in immunoreactivity reported in in all types of sourdough bread relative to the reference bread sample. The most amount of QQQPP allergen was determined at pH = 2 in wheat bread whether the sourdough was commercial or laboratory. The results were shown in Figure 2.

(b.) Intestinal disorders: When only half a physiological dose of pancreatin was used, the immunoreactivity of bread made with commercial sourdough (samples B and C) was slightly increased. The use of laboratory sourdough, however, did not elevate bread allergenicity beyond the level set by reference bread sample. The results were shown in Figure 3.

### 3.2. Inhibition of Enzymatic Conversion of Angiotensin

**Figure 4:**
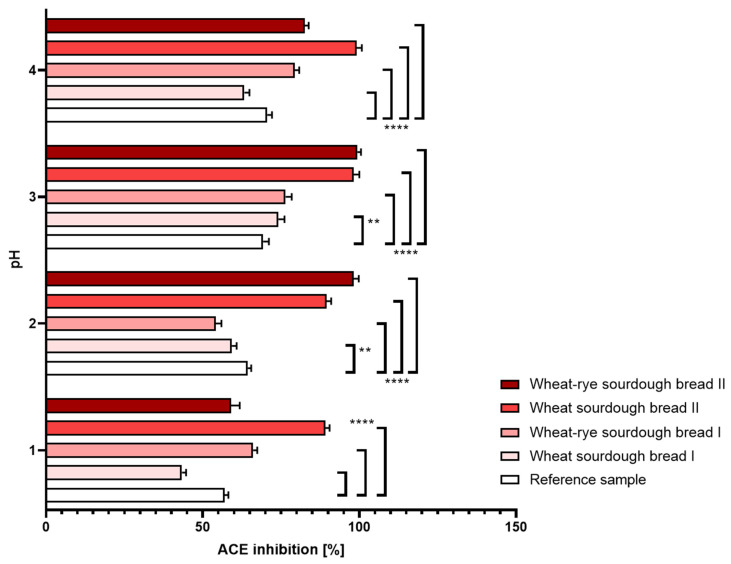
Inhibition of angiotensin-converting enzyme (±SD) determined in bread samples digested under different pH conditions corresponding to hyperacidity (pH = 1 and 2), physiological pH = 3 upon food consumption and hypoacidity at pH = 4. Statistical significance by 2-way ANOVA at ** *p* = 0.0014, 95% CI of diff. 1.770 to 8.430 (pH = 2),** *p* = 0.0022, 95% CI of diff. −8.230 to −1.570 (pH = 3), **** *p* < 0.0001, 95% CI of diff. at pH = 2: 10.27 to 16.93 (reference vs. wheat sourdough bread I), −12.43 to −5.770 (reference vs. wheat–rye sourdough bread I), −35.53 to −28.87 (reference vs. wheat sourdough bread II), 95% CI of diff. at pH = 2: 6.870 to 13.53 (reference vs. wheat–rye sourdough bread I), −28.43 to −21.77 (reference vs. wheat sourdough bread II), −37.13 to −30.47 (reference vs. wheat–rye sourdough bread II), 95% CI of diff. at pH = 3: −10.53 to −3.870 (reference vs. wheat–rye sourdough bread I), −32.23 to −25.57 (reference vs. wheat sourdough bread II), −33.43 to −26.77 (reference vs. wheat–rye sourdough bread II), CI of diff. at pH = 4: 3.568 to 11.03 (reference vs. wheat sourdough bread I), −12.14 to −5.462 (reference vs. wheat–rye sourdough bread I), −31.84 to −25.16 (reference vs. wheat sourdough bread II), −15.34 to −8.662 (reference vs. wheat–rye sourdough bread II).

**Figure 5:**
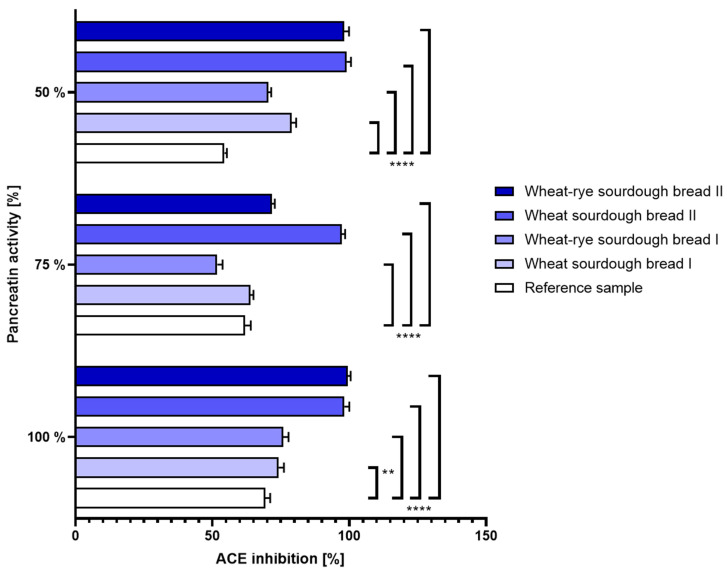
Inhibition of angiotensin-converting enzyme (±SD) determined in bread samples digested under different enzymatic conditions corresponding to 100%, 75% and 50% activity of pancreatin during intestinal phase of simulated digestion. Statistical significance by 2-way ANOVA at ** *p* = 0.0024, 95% CI of diff. −8.215 to −1.585; **** *p* < 0.0001, 95% CI of diff. at 100% pancreatin: −9.915 to −3.285 (reference vs. wheat–rye sourdough bread I), −32.21 to −25.59 (reference vs. wheat sourdough bread II), −33.41 to −26.79 (reference vs. wheat–rye sourdough bread II), 95% CI of diff. at 75% pancreatin: 6.785 to 13.41 (reference vs. wheat–rye sourdough bread I), −38.71 to −32.09 (reference vs. wheat sourdough bread II), −13.11 to −6.485 (reference vs. wheat–rye sourdough bread II), 95% CI of diff. at 50% pancreatin: −27.81 to −21.19 (reference vs. wheat sourdough bread I), −19.31 to −12.69 (reference vs. wheat–rye sourdough bread I), −48.01 to −41.39 (reference vs. wheat sourdough bread II), −47.01 to −40.39 (reference vs. wheat–rye sourdough bread II).

(a.) Gastric disorders: In the reference bread sample, digestion performed under different pH conditions did not translate into noticeable changes determined in the level of angiotensin conversion. Bread samples manufactured with laboratory sourdough (D and E) were marked by a significantly enhanced inhibition of ACE relative to reference bread as well as to the samples made with commercial sourdough. The reported effect was robust and not affected by either hypo- or hyperacidity conditions. The most pronounced inhibitory properties were seen to occur consistently under non-disturbed, physiological pH conditions. The results were shown in Figure 4.

(b.) Intestinal disorders: The same can be said about the ACE inhibition determined in bread samples when variable pancreatin activity was used during simulated digestion. At all tested enzyme doses, wheat and wheat–rye bread produced with laboratory sourdough displayed superior characteristics to its counterparts. The results were shown in Figure 5.

### 3.3. Inhibition of α-Amylase

**Figure 6:**
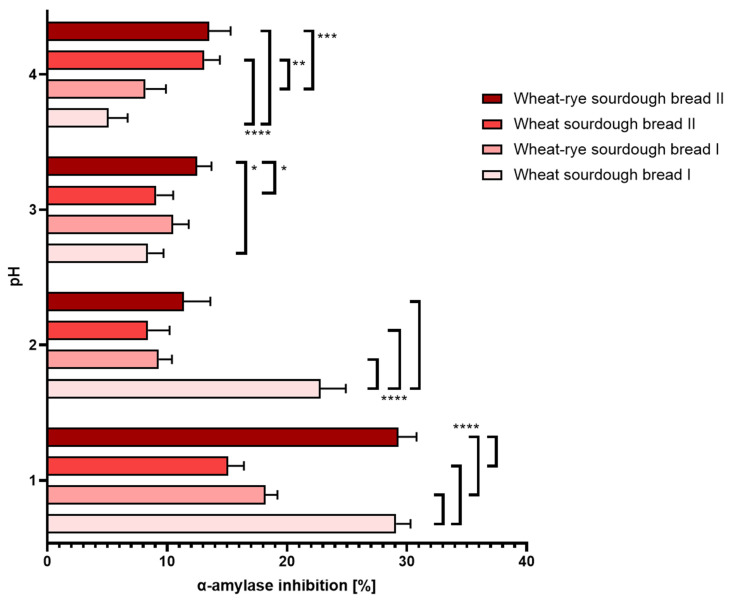
Inhibition of α-amylase (±SD) determined in bread samples digested under different pH conditions corresponding to hyperacidity (pH = 1 and 2), physiological pH = 3 upon food consumption and hypoacidity at pH = 4. Statistical significance by 2-way ANOVA at * *p* = 0.0478, 95% CI of diff. −6.775 to −0.02464 (wheat sourdough bread II vs. wheat–rye sourdough bread II); * *p* = 0.0125, 95% CI of diff. −7.475 to −0.7246 (wheat sourdough bread I vs. wheat–rye sourdough bread II); ** *p* = 0.0023, 95% CI of diff. −8.275 to −1.525; *** *p* = 0.0010, 95% CI of diff. −8.675 to −1.925; **** *p* < 0.0001, 95% CI of diff. at pH = 2: 7.525 to 14.28 (wheat sourdough bread I vs. wheat–rye sourdough bread I), 10.62 to 17.38 (wheat sourdough bread I vs. wheat sourdough bread II), −14.48 to −7.725 (wheat–rye sourdough bread I vs. wheat–rye sourdough bread II), −17.58 to −10.82 (wheat sourdough bread II vs. wheat–rye sourdough bread II), 95% CI of diff. at pH = 3: 10.12 to 16.88 (wheat sourdough bread I vs. wheat–rye sourdough bread I), 11.02 to 17.78 (wheat sourdough bread I vs. wheat sourdough bread II), 8.025 to 14.78 (wheat sourdough bread I vs. wheat–rye sourdough bread II), 95% CI of diff. at pH = 4: −11.77 to −4.226 (wheat sourdough bread I vs. wheat sourdough bread II), −12.17 to −4.626 (wheat sourdough bread I vs. wheat–rye sourdough bread II).

**Figure 7:**
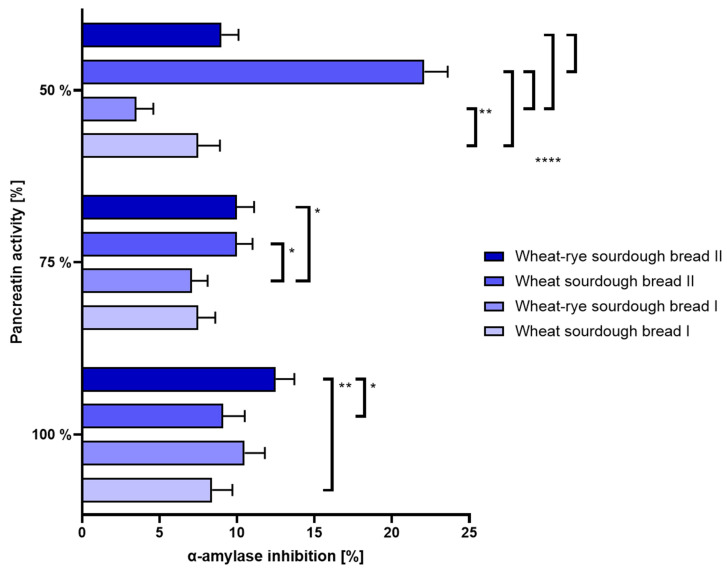
Inhibition of α-amylase (±SD) determined in bread samples digested under different enzymatic conditions corresponding to 100%, 75% and 50% activity of pancreatin during intestinal phase of simulated digestion. Statistical significance by 2-way ANOVA at * *p* = 0.0113, 95% CI of diff. −6.146 to −0.6544 (100%); * *p* = 0.0357, 95% CI of diff. −5.646 to −0.1544 (75%); ** *p* = 0.0021, 95% CI of diff. −6.846 to −1.354 (100%); ** *p* = 0.0026, 95% CI of diff. 1.254 to 6.746 (50%); **** *p* < 0.0001, 95% CI of diff.: −17.35 to −11.85 (wheat sourdough bread I vs. wheat sourdough bread II), −21.35 to −15.85 (wheat–rye sourdough bread I vs. wheat sourdough bread II), −8.246 to −2.754 (wheat–rye sourdough bread I vs. wheat–rye sourdough bread II), 10.35 to 15.85 (wheat sourdough bread II vs. wheat–rye sourdough bread II).

(a.) Gastric disorders: At physiological pH and above, all tested sourdough bread samples exhibited similar level of α-amylase inhibition. Overly acidic pH conditions amplified differences between the consecutive samples, but in every case, it largely elevated the extent of the inhibition. The results were shown in Figure 6.

(b.) Intestinal disorders: The mostly stable level of ACE inhibition at 100% and 75% pancreatin activity is thought to indicate no clear link between pancreatin and α-amylase activities. Changes in α-amylase activity reported at the lowest pancreatin concentration tested were not conclusive. The results were shown in Figure 7.

## 4. Discussion

It is a well-known fact that sourdough fermentation affects a number of bread dough properties, including gliadin and glutenin content, rheological properties or bread-making quality [34]. In a study by Fraberger et al., lactic acid bacteria showed a diverse capability of breaking down gliadin. Out of the 87 tested strains, 4% were reported as neutral, 52% had a moderate impact on gliadin, while 18% were described as having strong strain-dependent proteolytic activity [20]. In this work, we focused on how the health-promoting properties of bread can be modulated in the context of disorders that typically alter the biochemical environment in the gastrointestinal tract. It was postulated that it could be somewhat manipulated with the pre-designed choice of sourdough composition used at the stage of dough fermentation. Our findings revealed the advantageous effect of sourdough composed of *L. plantarum* BS, *L. brevis* 1269, and *L. sanfranciscensis* 20663 based on its gliadin digestion capabilities, which was referred to as *laboratory sourdough*, as opposed to commercially available sourdough. Having manufactured wheat and wheat–rye bread using the three selected strains of lactic acid bacteria, we obtained a product of clinical nutrition that could potentially exhibit desired health-promoting properties. To obtain a comprehensive insight into this subject, we tested three parameters that are largely decisive on how well the food is tolerated by the afflicted patients.

The most well-characterized forms of allergy to ingested wheat include wheat-dependent exercise-induced anaphylaxis (WDEIA), atopic dermatitis, anaphylaxis and urticaria. Wheat allergy can result in immunoglobulin E (IgE) and non-IgE mediated reactions, the former being immediate and life-threating [35]. Pentapeptide QQQPP is a peptide motif found in low molecular weight subunits of glutenin and a main IgE-binding epitope in hypersensitive patients [36]. It is therefore often selected by researchers as a marker to evaluate the level of immunogenicity of cereal-based foods and their overall safety. Our work has indicated a reduced immunoreactivity of sourdough bread digested under conditions occurring in the course of hypochlorhydria. This suggests its consumption could be more beneficial to the allergic to wheat compared to the bread, the production of which did not involve the use of sourdough. The specific composition of the sourdough became significant under the conditions of largely reduced pancreatin activity. While at 100% pancreatin activity, only one bread sample (produced with laboratory inoculum) displayed a somewhat decreased QQQPP content, the lower availability of pancreatin revealed a much clearer trend toward the reduced allergenicity of the sourdough bread. Bread samples baked with laboratory starter were the only ones where the allergenicity was consistently lower or, in just two cases, on par with reference control. Contrary to the commercial sourdough, the one proposed by our team prevented the reported increase in bread immunoreactivity. This finding may be of importance in the course of exocrine pancreatic insufficiency, coinciding with hypersensitivity to wheat.

Angiotensin is a peptide hormone that takes part in blood pressure regulation. Angiotensin-converting enzyme (ACE) performs a conversion of the inactive form of the hormone (Ang I) to angiotensin II (Ang II), which works to increase blood pressure by constricting the muscular walls of small arteries. ACE also degrades active bradykinin, the role of which is to increase blood vessel permeability and facilitate vasodilation. As a result, the blood pressure is elevated. ACE inhibitors are used to prevent hypertension and mitigate the risk of its consequence in the form of cardiovascular diseases [37]. Sourdough fermentation largely affects the nutritional value of the food by modulating the composition of bioactive compounds therein. Some of these compounds leave an imprint on the ACE inhibitory properties of bread, thus adding merit to its clinical use and its potential to supplement pharmaceutical medication. Our work highlighted the suitability of a sourdough bread for patients diagnosed with hypertension. Interestingly, these augmented ACE inhibitory properties were only observed in bread prepared from laboratory sourdough, suggesting the vital role played by specific strains selected for fermentation. Bread manufactured with starter proposed by our team was consistently marked with more pronounced ACE inhibitory properties regardless of pH. Only at the conditions of hyperacidity, a single sample was not statistically different from the reference. Other than that, a clear, statistically confirmed effect was reported. A similar pattern of results was evident under conditions of pancreatin deficiency where all samples inoculated with the laboratory starter were visibly more inhibitory to ACE than the reference with *p* < 0.0001. The robustness of data seen throughout the experiment leads us to recommend this type of bread to hypertensive patients without additional restrictions concerning their gastrointestinal health. Sourdough fermentation has also been attributed ACE inhibitory properties in a recent report where flour from *Triticum dicoccum* was tested. Peptides extracted from fermented flour exhibited 26.71% ACE inhibition compared to 13.82% by non-fermented sample [38]. In our research, we have shown that these properties persist despite thermal processing and they remain functional in a final product after the baking procedure.

The mechanism by which ACE inhibition was increased by bread extract has not been explored. It is, however, likely that it was due to the formation of inhibitory peptides, much like those described in related studies. ACE inhibitory activities were determined in digested faba bean flour and attributed to four peptides made up of 9–10 amino acid residues and showing IC_50_ values as low as 43 ± 1 μM and 100% inhibition at 10 mM. As opposed to commercial ACE inhibitors, the potency of which may be up to 300-fold higher, these peptides were shown to bind the enzyme outside the active site containing a zinc coordination motif. This means they are not competitive with the substrate. This fact largely hinders elucidation of the exact molecular mechanism of inhibition. Simulations point to the entrance of the active site cavity as a probable target of inhibitors binding, the act of which is believed to occur via hydrogen bonds and to limit the substrate entry and/or product exit from the active site cavity [39].

Molecules as small as penta- and tripeptides and 10-fold lower potency, respectively, were also reported in another study and deemed bioactive. The authors, however, identified them as reversible competitive inhibitory, meaning that they interact with the active site, but in the presence of a substrate, the effect depends on the concentration of the molecules [40]. The conclusion from both reports indicates that the inhibitory potency is largely affected by the number of hydrogen bonds that provide the stabilization of a complex structure. Shorter peptides with their limited binding capacity are at the same time those of higher IC_50_ values. Also, the catalytic center of ACE is more accessible for shorter peptides, which is likely why tripeptides were considered competitive, and nona- or decapeptides were considered non-competitive inhibitors.

ACE inhibitory peptides were also reported to be found in the hydrolyzed collagen of Kacang goat skin (IC_50_ 0.83 μg/mL) at a low molecular hydrolysate fraction [41] or in the hydrolysate of longan seeds. The latter were two decapeptides acting as non-competitive inhibitors. Also in this case, computational molecular docking simulation showed the interaction was H-bonds-dependent and occurred within non-active sites of ACE [42].

Reported consistent ACE inhibition could have potential clinical applications as well as limitations. The former, however, would be suggested as a support to the pharmaceutical therapy rather than the sole means of treatment. Specifically, sourdough bread has been a subject of many discussions in this context. Sourdough fermentation, among other processing conditions, can be adapted to modulate the glucose response to bread and lower the postprandial insulin response [43]. In a recent study, researchers reported on bread fermented with *S. cerevisiae* UFMG A-905 and its potential to prevent asthma. Microencapsulated live yeasts allowed for producing bread, the consumption of which resulted in less airway inflammation and lower levels of asthma biomarkers in mice [44]. In addition to adjusting the manufacturing procedure of bread itself, the product can be further fortified with supplementation of vitamins, minerals, fiber, proteins, and polyphenolic compounds, which add to its health-promoting properties [45]. The incorporation of prebiotics into bread could potentially affect the course of type 2 diabetes, heart disease, metabolic syndrome, and osteoporosis [46]. The stability and bioavailability of the bioactive compounds in fermented bread are their potential limiting factor, as is the case with prebiotic microbiota and their survival in a product that undergoes thermal processing and potentially long storage. Attempts to include dietary products as more than optional supplementation would require their meticulous characterization with this regard in view.

Pancreatic enzyme α-amylase is transported to and operates in the duodenal portion of the small intestine where it digests starch into short, linear oligosaccharides of maltotriose and maltose. A small portion of total amylase is produced in salivary glands. A minor concentration of the enzyme is in the blood serum, circulating to various organs [47]. Abnormally high or low levels of pancreatic amylase are associated with a 2–3 fold greater risk of developing pancreatitis and pancreatic cancer [48]. What is more, α-amylase is a drug target exploited in the treatment of diabetes. Its inhibition is conducive to disabling dietary carbohydrate digestion, which in turn decreases diet-dependent blood glucose rise [41]. Bread capable of inducing α-amylase inhibition would be a valuable supplementary tool against this disease. In our work, we observed an approximately 10% inhibition of alpha amylase in bread digested at pH = 3 regardless of the type of bread and sourdough used. Whether or not the consumption of sourdough bread could in any way contribute to modulating the enzyme activity remains speculative. No clear link could be inferred between alpha amylase inhibition and bread characteristics in the pH conditions tested. In a related paper, the authors have argued that α-amylase inhibitors present in gluten-containing foods are degraded during sourdough fermentation as the pH drops below 4, thereby presenting the fermentation process as promoting the hydrolysis of starch and proteins as well as enhancing the nutritional value of wheat [49]. This is somewhat contradictory to our findings where the highest rate of enzyme inhibition was reported in a strongly acidic environment. Also, questions were raised about the varieties used in the published research. To address this issue, 22 different wheat varieties were investigated to verify the change in the content of amylase inhibitors upon sourdough fermentation. Four fermented for 12 h indicated up to a 41% reduction in amylase inhibitors, and the results were significantly influenced by a variety of crops as well as their growing location [50]. The reported scatter between the samples of different variety and origin and the prolonged fermentation procedure could be a distinguishing factor when analyzing our results. A 1 h fermentation applied in this work could not suffice to introduce a significant extent of degradation of amylase inhibitors in tested samples. As different flours were used (wheat and rye–wheat) with no specific focus on the varieties of crops, different inhibitors content could be inherent to the samples, and a different extent of vulnerability to degradation could be attributed depending on the sourdough used for individual samples.

A multitude of technological factors affect the digestibility of bread, i.e., its amount of nutrients readily absorbed in the digestive tract [43]. With a higher intake of high-quality proteins, their health-promoting role becomes all the more valid. The presented approach to induce or to improve these properties in bread is based on the choice of specific microbial strains used at the stage of sourdough fermentation. The fermentation process is conducive to the synthesis or modification of bioactive compounds that would otherwise be absent from the final dietary product. In our research, the gliadin-degrading microbiota selected as a sourdough starter were capable of promoting the anti-hypertensive properties. It occurred due to the emergence of chemical molecules that were able to interact with ACE or regulate its activity indirectly. To further fine tune the quality of bread dough, other tools can also be exploited. Flour properties like amylolytic capacity and bread-making properties can be exposed to ultrasound-assisted drying techniques [44] or other forms of physico-chemical treatment, which could potentially alter the final bioproduct toward an even more desirable profile.

## 5. Conclusions

Based on the results of the presented research, we can postulate that functional food can be considered for use to reinforce the clinical treatment of hypertension. Sourdough bread is a dietary staple with a highly affordable production cost and high nutritional value, which makes it a good candidate for the role of therapeutic support. Some of its properties, including gluten content, can be altered depending on the composition of inoculum. Lower gluten levels not only affect the structural and organoleptic profile of bread, they also lower the dietary exposure of patients afflicted with allergies and intolerances. Other than that, our research confirmed that fermentation is an especially vital part of bread manufacturing not only in the context of gatekeeping symptoms of dietary disorders but also as a tool to obtain proactive health-promoting functionality. Bread with decreased gluten content produced with a specifically selected sourdough microbiota is capable of mitigating hypertension. Our research indicates that the consumption of bread produced with sourdough composed of gliadin-degrading strains of bacteria (*L. plantarum* BS, *L. brevis* 1269, *L. sanfranciscensis* 20663) is likely to stimulate the inhibition of angiotensin-converting enzyme. As such, it contributes to impairing the physiological mechanism responsible for increasing blood pressure. Inhibited ACE translates into less constricted muscular walls of small arteries, which in turn is conducive to relieving hypertension. The reported effect is robust and suitable for patients afflicted with additional disorders of gastrointestinal tract that compromise the digestion process. In this study, we performed an analysis on wheat and wheat–rye bread. For researchers interested in taking up this subject, it is encouraged to broaden the research with other types of bakery products. Also, for elucidation of the mechanism involved in the reported outcome, it would be interesting to see the experimental effect recreated in vitro using human cell lines with the use of angiotensin and ACE-specific antibodies.

## Figures and Tables

**Table 1:** Simulated salivary fluid (SSF).

Reagent	Concentration [mol/dm^3^]	Volume [mL]
KCl	0.5	15.1
KH_2_PO_4_	0.5	3.7
NaHCO_3_	1	6.8
MgCl_2_(H_2_O)_6_	0.15	0.5
(NH_4_)_2_CO_3_	0.5	0.06
Distilled water		Up to 400

**Table 2:** Simulated gastric fluid (SGF).

Reagent	Concentration [mol/dm^3^]	Volume [mL]
KCl	0.5	6.9
KH_2_PO_4_	0.5	0.9
NaHCO_3_	1	12.5
NaCl	2	11.8
MgCl_2_(H_2_O)_6_	0.15	0.4
(NH_4_)_2_CO_3_	0.5	0.5
Distilled water		Up to 400

**Table 3:** Simulated intestinal fluid (SIF).

Reagent	Concentration [mol/dm^3^]	Volume [mL]
KCl	0.5	6.8
KH_2_PO_4_	0.5	0.8
NaHCO_3_	1	42.5
MgCl_2_(H_2_O)_6_	0.15	1.1
NaCl	2	9.6
Distilled water		Up to 400

## Data Availability

The original contributions presented in the study are included in the article, further inquiries can be directed to the corresponding authors.
